# *Poncirus trifoliata* vs. *Citrus junos* rootstocks: reshaping lemon rhizosphere microecology through microbial and metabolic reprogramming

**DOI:** 10.3389/fmicb.2025.1650631

**Published:** 2025-09-24

**Authors:** Chunrui Long, Xiaomeng Fu, Qingjiang Wu, Shaohua Wang, Xianyan Zhou, Jiamei Mao, Lina Guo, Wenbin Shi, Hongxia Yang, Tiankun Yang, Yuxia Du, Jianqiang Yue, Dongming Wu, Hongming Liu

**Affiliations:** ^1^Institute of Tropical and Subtropical Cash Crops, Yunnan Academy of Agricultural Sciences, Baoshan, China; ^2^Institute of Horticultural Sciences, Jiangxi Academy of Agricultural Sciences, Nanchang, China; ^3^Ministry Key Laboratory of Low-carbon Green Agriculture in Tropical Region of China, Haikou, China; ^4^Hainan Key Laboratory of Tropical Ecocirculing Agriculture, Institute of Environment and Plant Protection, Chinese Academy of Tropical Agricultural Sciences, Haikou, China

**Keywords:** lemon rootstocks, rhizosphere microbiota, metabolomic profiling, rootstock-microbe interactions, soil microecology

## Abstract

**Introduction:**

Trifoliate orange (*Poncirus trifoliata*L. Raf) and “Ziyang Xiangcheng” (Citrus junos Sieb. ex Tanaka) are the predominant rootstocks for lemon production in China, exhibiting distinct adaptations to soil pH and differential impacts on plant resilience. As pivotal mediators of scion-soil interactions, rootstocks have emerged as key research targets for their regulatory potential in rhizosphere microbial communities and metabolites.

**Methods:**

Pot-cultured systems were established with lemon (*Citrus* × *limon* “Eureka”) saplings grafted onto trifoliate orange (PTL) and “Ziyang Xiangcheng” (CJL) rootstocks. Integrated metagenomic and GC-MS metabolomic approaches were employed to analyze rhizosphere microbial communities and metabolites.

**Results:**

The results demonstrated no significant difference in rhizospheric microbial α-diversity (richness) between PTL and CJL, although PTL exhibited higher evenness. *β*-Diversity and LEfSe analysis revealed significant structural divergence in communities. A total of 15 differentially enriched genera across three phyla were identified, among which *Pseudomonas*, *Cupriavidus*, and *Burkholderia* in CJL, along with *Sphingobium* in PTL, exhibited strong effects. Random forest modeling identified 15 key differential metabolites, with 4 significantly upregulated in CJL and 11 in PTL. Microbial-metabolite correlation and GSEA analysis uncovered 10 core pathways involving genetic information processing, energy metabolism, environmental adaptation, and disease resistance mechanisms. Soil analysis showed CJL significantly surpassed PTL in organic matter content, catalase activity and plant height, whereas PTL exhibited superior cellulase, sucrase and urease activities. Mechanistically, PTL appears to recruit *Pseudomonas mediterranea* via 1-Monostearin secretion to activate glycerolipid metabolism, enhancing drought tolerance. Its caffeate and salicyl alcohol-*β*-glucoside secretions potentially mobilize *Sphingobium* and *Ensifer* adhaerens to regulate amino sugar metabolism, promoting carbon sequestration and root defense. Conversely, CJL likely employs L-alanine exudation to recruit Pseudomonas putida, triggering exopolysaccharide biosynthesis through arginine-proline metabolism as a key tolerance mechanism (such as drought tolerance and alkali tolerance)

**Discussion:**

The findings elucidate rootstock-specific modulation of rhizosphere microecosystems, highlighting distinct microbial-metabolite interactions and tolerance mechanisms. These results provide theoretical support for precision rootstock selection and microbiome engineering to advance sustainable citrus production.

## Graphical abstract

Rootstock-mediated shaping of lemon rhizosphere microecology: microbial and metabolic reprogramming strategies.
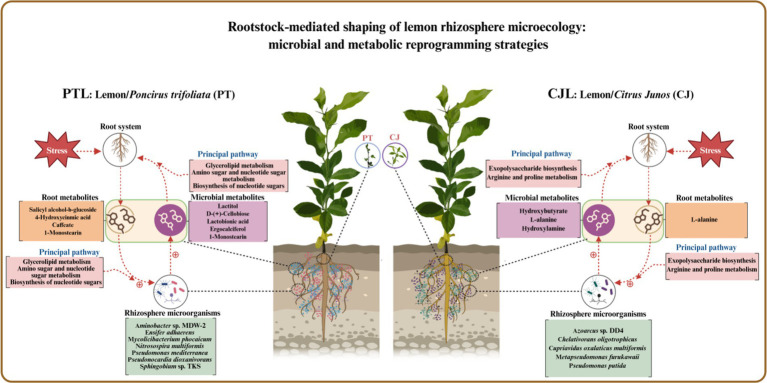

## 1 Introduction

Lemon (*Citrus limon*) is a globally important economic fruit tree whose fruits contain abundant bioactive compounds including vitamin C, organic acids and flavonoids, with wide applications in food, pharmaceutical and cosmetic industries. In commercial production, grafted lemon trees predominate, with rootstocks significantly influencing fruit quality, yield, as well as tree adaptability and stress resistance (Goldschmidt, 2014). However, sustainable lemon production faces major challenges including soil sickness, soil-borne diseases and inefficient nutrient utilization. Conventional cultivation relying on chemical fertilizers and pesticides not only increases costs but also exacerbates soil acidification, reduces microbial diversity and degrades ecosystem services (Nair and Ngouajio, 2012). Consequently, green production models based on rhizosphere microecological regulation have emerged as a research focus. As the critical interface mediating interactions between the scion and the soil environment, the regulatory potential of rootstocks over rhizosphere microbial communities and metabolites is garnering increasing attention (Liu et al., 2018; Wang et al., 2014; Ruan et al., 2020). Grafting creates a novel rootstock-scion composite whose root characteristics are rootstock-determined (Goldschmidt, 2014). Plants reshape their rhizosphere through root architecture (RellánÁlvarez et al., 2016; Ragland et al., 2024), exudate composition (Mommer et al., 2016) and stress signaling (Tan et al., 2024), influencing microbial colonization and functionality (Shi et al., 2025; Ragland et al., 2024), thereby regulating nutrient uptake, disease resistance and stress tolerance (Hodge et al., 2009). For instance, studies demonstrate that wild soybean under salt stress recruits *Pseudomonas* bacteria via root-secreted purine metabolites like xanthine, mitigating salt-induced growth inhibition (Zheng et al., 2024). Similarly, maize root exudates attract *Bacillus amyloliquefaciens* OR2-30, which inhibits conidial formation and germination in *Fusarium graminearum*, induces reactive oxygen species (ROS) production, and triggers hyphal cell death (Xie et al., 2022). In potato monoculture systems, root secretion of nobiletin recruits *Pantoea* sp. MCC16, a high auxin (IAA)-producing strain, restoring root functional traits and alleviating replant disease to enhance yield (Ma et al., 2025). Furthermore, ginger (*Zingiber officinale*) root exudates, including compounds like sinapyl alcohol and 6-gingerol, significantly enhance the colonization and proliferation of *Burkholderia* species in the chrysanthemum (*Chrysanthemum morifolium*) rhizosphere, strengthening suppression against *Fusarium* wilt (Zhu et al., 2025).

Rootstocks modify and adapt to soil environments by actively recruiting or suppressing specific microorganisms, yet current research predominantly focuses on single rootstock varieties or single-omics approaches. For instance, a study comparing four citrus rootstocks under varying phosphorus levels revealed that low-P-tolerant genotypes significantly increased rhizospheric organic acid secretion and enhanced soil biological activity under phosphorus starvation (Luo and Fan, 2014). Similarly, Sun et al. (2017) demonstrated higher genus-level microbial abundance in *Citrus willsonii* rootstock compared to *Poncirus trifoliata* when grafted with *Satsuma mandarin*, primarily enriching iron-uptake-associated microbiota. Further evidence from Raiesi et al. (2022) indicates that rootstock identity governs rhizosphere phosphorus fraction dynamics and biochemical properties induced by root activity. Conversely, rhizosphere microorganisms reciprocally influence rootstocks—while AMF (Arbuscular Mycorrhizal Fungi) effects on citrus drought resilience (Zou et al., 2013a; Wu et al., 2017), salt tolerance (Zou et al., 2013b), growth promotion (Chen et al., 2017), and disease resistance (Zhang, 2019; Watanarojanaporn et al., 2011) are documented, impacts of other microbiota (e.g., Actinobacteria, Pseudomonadota) remain understudied. Critically, the molecular mechanisms through which diverse rootstocks shape citrus rhizosphere microbiomes and metabolomes remain elusive, particularly at multi-omics integration levels, hindering theoretical advances in rootstock-microbe interactions and precision management development. Moreover, prevailing rootstock evaluation systems rely excessively on phenotypic parameters (e.g., grafting success rate, yield), failing to incorporate rhizospheric microbial functional traits into selection criteria, which compromises breeding precision.

Trifoliate orange (*Poncirus trifoliata* L. Raf) and Ziyang Xiangcheng (*Citrus junos* Sieb. ex Tanaka) are the most widely employed superior rootstocks in lemon production. *Poncirus trifoliata* rootstock offers advantages including strong graft compatibility, early high yield, drought tolerance, cold hardiness, and resistance to foot rot; it tolerates strongly acidic soils but exhibits poor alkaline tolerance. Conversely, *Citrus junos* rootstock is characterized by a vigorous root system, robust tree growth, high productivity, and cold tolerance, displaying exceptional alkaline tolerance but sensitivity to strongly acidic conditions (Zheng N. et al., 2021). To elucidate the mechanisms by which these distinct rootstocks influence rhizosphere microbes and metabolites, we conducted a two-year pot experiment. This study utilized lemon grafted seedlings on these two rootstocks, employing integrated rhizosphere soil metagenomics and metabolomics to characterize differences in microbial community structure and metabolic profiles, thereby investigating rootstock-mediated functional regulation of the rhizosphere microecology. Key scientific questions addressed include: (1) What microbial signatures are enriched in the rhizosphere of each rootstock type? (2) How do rootstock-specific rhizosphere metabolite enrichment patterns differ? (3) Do key microbial taxa and metabolite modules synergistically contribute to the stress resistance (or tolerance) traits of lemon rootstocks? Through integrated omics analysis, core functional rhizosphere metabolites and microbial biomarkers will be identified. On the one hand, it provides reference for screening rootstocks with desirable root traits for lemon cultivation, and on the other hand, it offers theoretical support for developing soil-applied biological agents and biopesticides, thereby promoting the shift of the lemon industry toward ‌resource-efficient and environmentally friendly practices‌.

## 2 Materials and methods

### 2.1 Experimental site and experimental design

The experiment was conducted in 2022 at the Institute of Tropical and Subtropical Cash Crops, Yunnan Academy of Agricultural Sciences (25°8′10″N, 99°10′53″E; Baoshan, China), a subtropical dry-hot valley climate zone at 700 m altitude with mean annual temperature of 21.5 °C and ~750 mm precipitation, using sandy loam soil (pH 6.5–7.0) containing 40.04 g·kg^−1^ organic carbon, 69.02 g·kg^−1^ organic matter, 2.60 g·kg^−1^ total N, 182.30 mg·kg^−1^ alkali-hydrolyzable N, 20.67 g·kg^−1^ total K, 366.29 mg·kg^−1^ available K, 1.14 g·kg^−1^ total P, and 118.34 mg·kg^−1^ available P.

This study implemented two treatments: (1) Eureka lemon (*Citrus limon* “Eureka”) grafted onto *Citrus junos* rootstock (CJL) and (2) Eureka lemon grafted onto *Poncirus trifoliata* rootstock (PTL), with 18 potted replicates per treatment (pot dimensions: 25 cm diameter × 40 cm height; 5-gallon capacity) using uniformly vigorous rootstocks (height: 10–15 cm) that were root-washed and transplanted; grafting with healthy Eureka lemon scions commenced when rootstock stem diameter reached 0.5–0.8 cm, followed by training to retain a single main stem pruned at 30 cm height with lateral shoots pinched to 10–15 cm length, under consistent cultivation conditions featuring monthly compound fertilizer application (N-P_2_O_5_-K_2_O = 17:17:17; 5 g per plant), quarterly foliar micronutrient-enriched water-soluble fertilizers, and standardized pest/disease control, culminating in rhizosphere soil collection from all 36 biological replicates (18 per treatment) after 24 months of cultivation.

### 2.2 Soil sampling collection

Rhizosphere soils were carefully collected by brushing adherent soil from root systems, followed by removal of gravel and residual roots. The soil sample was divided into two parts after being screened by 2 mm. One part of the soil was stored at −80 °C for metagenome sequencing, and the other part was stored at −80 °C for GC-MS non-target metabolomics detection after vacuum freeze-drying.

### 2.3 Metagenomic sequencing and analysis

Total microbial genomic DNA was extracted from rhizosphere soils using the CTAB method and quantified using an Agilent 5,400 Fragment Analyzer for concentration, integrity (DV50 > 20 kb), and purity (A260/280 ratio 1.8~2.0). Qualified DNA was fragmented into 350 bp inserts using a Covaris M220 Focused-ultrasonicator (Woburn, MA, United States) for library construction. Libraries were diluted to 1.5 nM using Qubit 2.0 Fluorometric Quantitation (Thermo Fisher Scientific), validated for insert size distribution via Agilent 2100 Bioanalyzer, and quantified by qPCR (Kapa Biosystems). Paired-end sequencing (2 × 150 bp) was performed on the Illumina NovaSeq 6000 platform (San Diego, CA, United States) by Wekemo Tech Group Co., Ltd. (Shenzhen, China).

Raw reads underwent quality control using FastQC (v0.12.0), with subsequent removal of adapter sequences and host-derived reads using KneadData (v0.12.0) (minimum Phred score: 20). Taxonomic profiling was performed using Kraken2 with the Standard Plus Protozoa/RefSeq database, followed by abundance estimation via Bracken (v2.8). Functional annotation employed HUMAnN3 (v3.6.0) with DIAMOND alignment against UniRef90 and EggNOG v5.0 databases to quantify orthologous gene families (KEGG pathways) and metabolic modules. All analyses used default parameters unless specified.

### 2.4 Untargeted GC-MS metabolomics

Frozen lyophilized soil samples (50 ± 2.5 mg) were homogenized in 2 mL microtubes with 0.5 mL of pre-chilled (−20 °C) acetonitrile:isopropanol (3:3:2, v/v/v) and 3–4 zirconium beads (2 mm). Tissues were disrupted using a high-throughput grinder (30 Hz, 20 s grinding/10 s pause, 8 cycles) followed by ice-water bath ultrasonication (5 min). After adding an additional 0.5 mL of extraction solvent, samples were re-sonicated (5 min) and centrifuged (12,000 × g, 2 min, 4 °C). Supernatants (500 μL) were concentrated to dryness via vacuum centrifugation (Christ RVC 2–25, 8~10 h) and reconstituted in 80 μL methoxyamine hydrochloride (20 mg/mL in pyridine) for 60 min derivatization at 60 °C. Subsequently, 100 μL BSTFA-TMCS (99:1) was added, vortex-mixed (30 s), and incubated at 70 °C for 90 min. Derivatized extracts were centrifuged (14,000 × g, 3 min), and supernatants (90~100 μL) were transferred to autosampler vials for GC-TOF MS analysis within 24 h.

Chromatographic separation was performed on an Agilent 7890B system equipped with a DB-5MS capillary column (30 m × 250 μm, 0.25 μm film; Agilent J&W Scientific) under helium carrier gas (1 mL/min constant flow). Injection volume was 1 μL in split mode (1:10 ratio) with inlet temperature at 280 °C. Oven temperature program: initial 50 °C (0.5 min hold), ramped at 15 °C/min to 320 °C (9 min hold). Mass spectrometry utilized an Agilent 7200 Q-TOF with electron ionization (−70 eV) in full-scan mode (m/z 50–600, 10 spectra/s). Interface and ion source temperatures were maintained at 320 °C and 230 °C, respectively, with 3 min solvent delay. All metabolomic profiling was conducted by Wekemo Tech Group Co., Ltd. (Shenzhen, China).

### 2.5 Determination of soil physicochemical properties and enzyme activities

Total nitrogen (TN) was quantified by the Kjeldahl method: Soil samples were digested with concentrated H_2_SO_4_ and catalyst to convert organic nitrogen to ammonium-N, followed by steam distillation, boric acid absorption, and titration with HCl standard solution. Alkali-hydrolyzable nitrogen (AN) was measured via alkali diffusion: Samples were hydrolyzed with 1.8 mol·L^−1^ NaOH at 40 °C for 24 h, with liberated NH_3_ absorbed in boric acid for titration. Organic carbon (OC) was determined by K_2_Cr_2_O_7_ oxidation-external heating: Organic matter was oxidized with 0.8 mol·L^−1^ K_2_Cr_2_O_7_-H_2_SO_4_ at 170–180 °C, and residual K_2_Cr_2_O_7_ titrated with FeSO_4_ standard solution. Organic matter (OM) content was calculated as OC × 1.724. All analyses included ‌triplicate measurements with certified reference materials for quality control.

Cellulase activity was assayed by DNS method: 5.0 g fresh soil reacted with 1% carboxymethylcellulose sodium (CMC-Na) in acetate buffer (pH 5.0) at 50°C for 24 h; reactions were terminated with 3,5-dinitrosalicylic acid, and reducing sugars quantified at 540 nm (units: μmol glucose g^−1^ 24 h^−1^). Invertase activity followed Hoffmann: 2.0 g soil incubated with 8% sucrose in phosphate buffer (pH 6.5) at 37 °C for 24 h, with glucose yield measured (units as above). Catalase activity used KMnO_4_ titration: 5.0 g soil reacted with 0.3% H_2_O_2_ at 25 °C for 20 min, and residual H_2_O_2_ titrated with 0.1 mol·L^−1^ KMnO_4_ (units: mL KMnO_4_ consumed g^−1^ 20 min^−1^). Urease activity employed indophenol blue colorimetry: 5.0 g soil incubated with 10% urea in citrate buffer (pH 6.7) at 37 °C for 24 h, with NH_4_^+^-N production quantified (units: μg NH_4_^+^-N g^−1^ 24 h^−1^). All enzymatic assays were performed in triplicate with substrate-free controls and standard curve calibration.

### 2.6 Plant height measurement

Plant height was measured using a ruler with 1 mm precision, recording the vertical distance from the soil surface at the root collar to the apical meristem. Three replicate measurements were taken per plant.

### 2.7 Statistical analyses

Soil physicochemical properties and enzyme activities were analyzed using one-way ANOVA, with significant differences (*p* < 0.05) determined by Duncan’s multiple range test for post-hoc comparisons. Plant height data underwent multiple comparisons through the Kruskal-Wallis test (non-parametric method) for single-factor designs.

Integrated multi-omics analyses were performed following standardized workflows to ensure reproducibility and biological relevance. Metagenomic raw sequences underwent quality control using KneadData (Trimmomatic for adapter removal; Bowtie2 for host DNA depletion) with FastQC validation of pre- and post-processing data quality (Bolger et al., 2014; Langmead and Salzberg, 2012). Taxonomic profiling was conducted via Kraken2 with a customized microbial database (incorporating bacterial, fungal, archaeal, and viral genomes from NCBI and RefSeq), refined through Bracken for species-level abundance quantification (Wood and Salzberg, 2014; Lu et al., 2017; Mandal et al., 2015; Brum et al., 2015). Functional annotation was implemented in HUMAnN3 using the UniRef90 database, with metabolic pathway abundance profiles generated via DIAMOND alignment (e-value ≤ 1e − 5) (Segata et al., 2011; Zhu et al., 2010; Kim et al., 2016; Franzosa et al., 2018). Antibiotic resistance genes were identified through DIAMOND alignment against the CARD database (bit-score > 60).

Microbial community analyses included beta-diversity assessment (Bray–Curtis dissimilarity), ordination analyses (PCoA/NMDS), and LEfSe biomarker detection (LDA score > 2) (Franzosa et al., 2018; Villar et al., 2015). Metabolomics data were processed using the MetaboAnalyst R package in R (Chong and Xia, 2018), encompassing batch effect correction, OPLS-DA for biomarker selection (VIP > 1.5), and mummichog-based pathway enrichment analysis (FDR < 0.1). Multi-omics integration involved data normalization (probabilistic quotient normalization for metabolites; Hellinger transformation for microbial features), followed by correlation analyses (sparse canonical correlation analysis sCCA, random forest) and joint pathway mapping via mummichog2-based KEGG enrichment. All statistical inferences were adjusted using the Benjamini-Hochberg FDR correction method.

## 3 Results

### 3.1 Analysis of rhizosphere soil microbial diversity in lemon trees with two types of rootstocks

Metagenomic sequencing of 36 samples generated a total of 843,080,544 raw sequences. After quality control, 778,709,052 valid sequences were obtained. The Q30 values for both raw and valid sequences in each sample exceeded 85%, indicating that the data quality was satisfactory and the sequencing depth was sufficient to reflect the microbial community structure in each sample.

**Figure 1:**
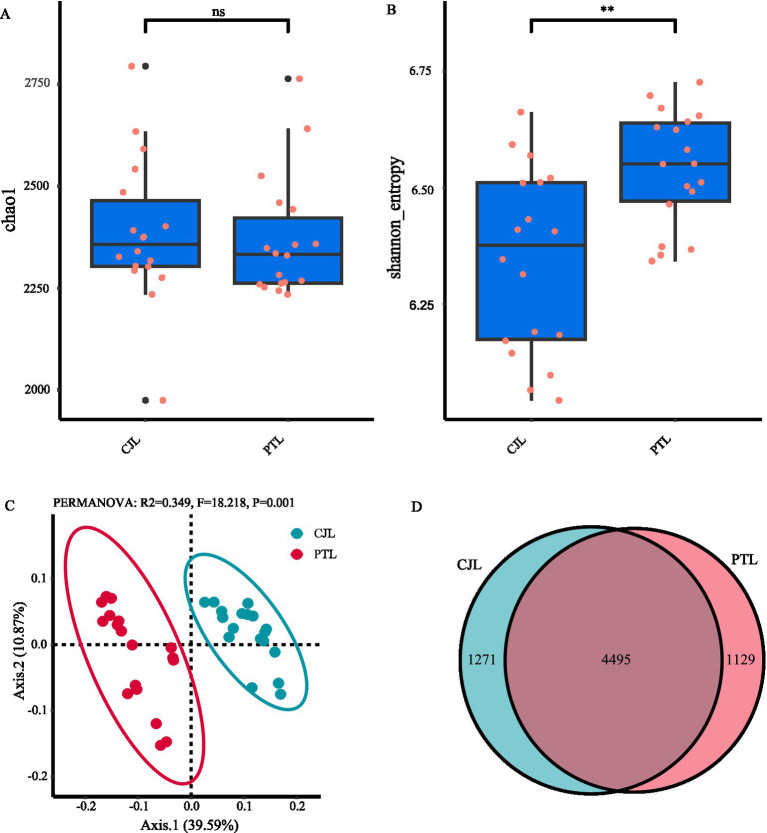
Diversity analysis of rhizosphere soil microbial communities in lemon trees with two types of rootstocks. **(A)** Chao1 index (richness estimator) comparison between CJL (*Citrus junos* rootstock) and PTL (*Poncirus trifoliata* rootstock). **(B)** Shannon index (diversity estimator) showing significant differences (^**^p < 0.01, Wilcoxon test). **(C)** Principal coordinate analysis (PCoA) based on Bray-Curtis distance, with PERMANOVA confirming significant compositional divergence (*R^2^ = 0.349*, *p = 0.001*). **(D)** Venn diagram illustrating shared (4495) and unique OTUs (CJL: 1,271; PTL: 1,129).

To assess the microbial community diversity, we utilized the Chao1 index, which reflects community richness, and the Shannon index, which accounts for both richness and evenness. The results showed that there was no significant difference in the Chao1 index between the rhizosphere soil microbial communities of the two rootstock lemons (Figure 1A). However, the Shannon index was significantly higher in PTL compared to CJL (Figure 1B; *p* < 0.01). Beta diversity analysis of the rhizosphere soil microbial communities of the two rootstocks was conducted using principal coordinate analysis (PCoA). The results revealed that the first principal component (Axis 1) explained 39.59% of the variation, while the second principal component (Axis 2) explained 10.87% (Figure 1C). The microbial community compositions of the two groups were significantly separated, indicating that the rootstock types affects the structure and composition of the rhizosphere soil microbial community. A Venn diagram further illustrated the species overlap between the two groups. The results showed that CJL and PTL shared 4,495 species, with 1,271 species unique to CJL and 1,129 species unique to PTL (Figure 1D).

### 3.2 Analysis of rhizosphere soil microbial community composition in lemon trees with two types of rootstocks

**Figure 2:**
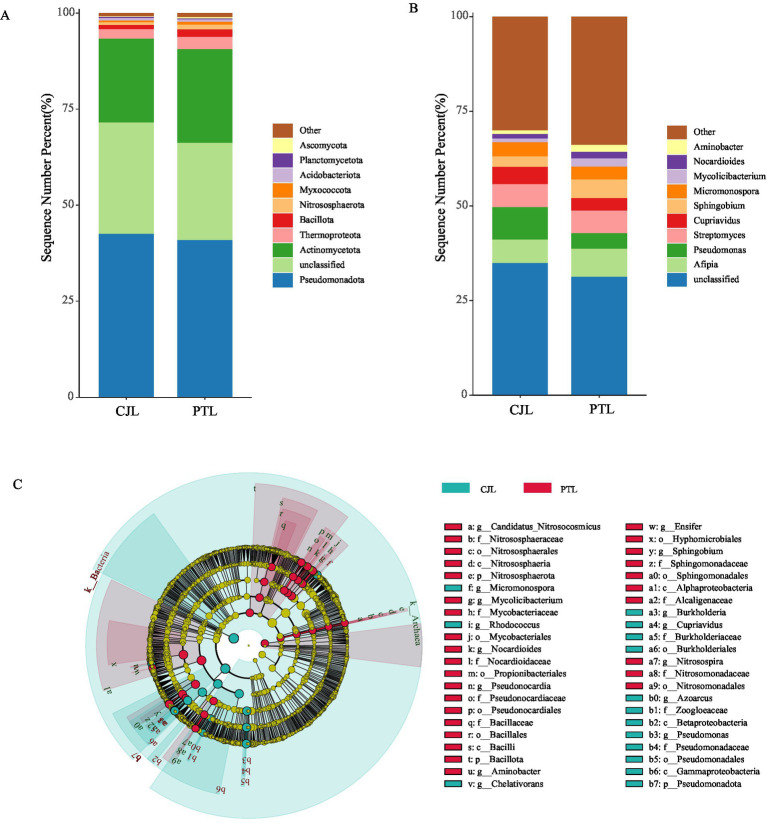
Microbial community abundance and group differences. **(A)** Relative abundance at the phylum level (top 10). **(B)** Relative abundance at the genus level (top 10). **(C)** LEfSe analysis (LDA score > 3.5) showing significantly different microbial taxa.

The metagenomic sequencing identified 96, 147, 298, 641, 1,798, and 6,895 taxonomic groups from phylum to species level, respectively. To investigate the microbial composition, we plotted the relative abundance of the top 10 microbial phyla in both treatments (Figure 2A). The results revealed that *Pseudomonadota*, *Actinomycetota*, and *Thermoproteota* were highly abundant in both rootstock rhizosphere soil microbial communities, with *Pseudomonadota* accounting for over 40% and *Actinomycetota* exceeding 20% average relative abundance in both treatments. Together, these three phyla constituted more than 65% of the total relative abundance, indicating their dominance. At the genus level, the top 10 microbial genera in both treatments included *Afipia*, *Pseudomonas*, *Streptomyces*, *Cupriavidus*, *Sphingobium*, *Micromonospora*, *Mycolicibacterium*, *Nocardioides*, *Aminobacter*, and unclassified genus (Figure 2B).

Building upon LEfSe analysis with a significance threshold (LDA score > 3.5), differentially abundant microbes were predominantly identified within three phyla: Actinomycetota, Pseudomonadota, and Nitrososphaerota (Figure 2C). Specifically, CJL-associated microbiota comprised seven enriched genera: *Micromonospora*, *Rhodococcus*, *Chelativorans*, *Burkholderia*, *Cupriavidus*, *Azoarcus,* and *Pseudomonas*. In contrast, PTL-associated microbiota revealed eight enriched genera: *Nitrosospira*, *Sphingobium*, *Ensifer*, *Aminobacter*, *Pseudonocardia*, *Nocardioides*, *Mycolicibacterium*, and *Candidatus Nitrosocosmicus*. Notably, four key taxa exhibited significantly higher discriminatory power (LDA score > 4): *Pseudomonas* (class Gammaproteobacteria), *Cupriavidus* (class Betaproteobacteria), and *Burkholderia* (class Betaproteobacteria) enriched in CJL; and *Sphingobium* (class Alphaproteobacteria) enriched in PTL. These high-impact microorganisms were consequently prioritized as focal differential populations.

### 3.3 Potential functional pathways of soil microorganisms in the rhizosphere of lemon trees with two types of rootstocks

**Figure 3:**
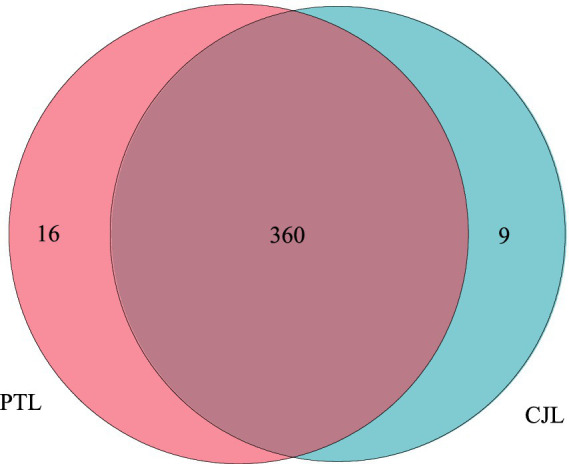
Venn diagram of annotated metabolic pathways. The 16 unique pathways in PTL were: (1) Metabolism; Glycan biosynthesis and metabolism; Glycosphingolipid biosynthesis-ganglio series. (2) Metabolism; Metabolism of terpenoids and polyketides; Type I polyketide structures. (3) Environmental Information Processing; Signal transduction; MAPK signaling pathway. (4) Organismal Systems; Immune system; Chemokine signaling pathway. (5) Cellular Processes; Transport and catabolism; Autophagy-other. (6) Organismal Systems; Development and regeneration; Axon guidance. (7) Environmental Information Processing; Signal transduction; VEGF signaling pathway. (8) Organismal Systems; Development and regeneration; Osteoclast differentiation. (9) Cellular Processes; Cellular community-eukaryotes; Signaling pathways regulating pluripotency of stem cells. (10) Organismal Systems; Immune system; Toll-like receptor signaling pathway. (11) Organismal Systems; Immune system; Toll and Imd signaling pathway. (12) Organismal Systems; Immune system; Natural killer cell mediated cytotoxicity. (13) Organismal Systems; Immune system; B cell receptor signaling pathway. (14) Organismal Systems; Immune system; Fc epsilon RI signaling pathway. (15) Organismal Systems; Immune system; Fc gamma R-mediated phagocytosis. (16) Human Diseases; Endocrine and metabolic disease; AGE-RAGE signaling pathway in diabetic complications. The 9 unique pathways in CJL were: (1) Environmental Information Processing; Signal transduction; NF-kappa B signaling pathway. (2) Environmental Information Processing; Signal transduction; Notch signaling pathway. (3) Environmental Information Processing; Signal transduction; Hedgehog signaling pathway-fly. (4) Environmental Information Processing; Signal transduction; TGF-beta signaling pathway. (5) Organismal Systems; Environmental adaptation; Circadian rhythm-plant. (6) Human Diseases; Endocrine and metabolic disease; Maturity onset diabetes of the young. (7) Human Diseases; Substance dependence; Morphine addiction. (8) Human Diseases; Substance dependence; Nicotine addiction. (9) Human Diseases; Cancer_specific types; chronic myeloid leukemia.

Comparison with the Kyoto Encyclopedia of Genes and Genomes (KEGG) database revealed that 385 Level 3 metabolic pathways were annotated across all samples (Figure 3). Among these, 369 pathways were detected in CJL and 376 in PTL, with 9 and 16 unique pathways exclusive to CJL and PTL, respectively (Figure 3). Screening identified one rhizosphere-relevant pathway in CJL: Organismal Systems > Environmental Adaptation > Circadian rhythm-plant. Three pathways were identified in PTL: (1) Metabolism > Metabolism of Terpenoids and Polyketides > Type I polyketide structures; (2) Environmental Information Processing > Signal Transduction > MAPK signaling pathway; (3) Cellular Processes > Transport and Catabolism > Autophagy-other.

**Figure 4:**
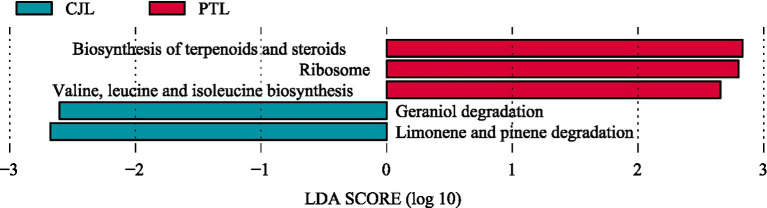
LEfSe analysis of metabolic pathways. Displaying the significantly different metabolic pathways between CJL and PTL, with LDA scores indicating the magnitude of difference.

LEfSe analysis (LDA score > 2) identified 52 metabolic pathways with statistically significant intergroup differences. Thirty pathways were significantly enriched in the CJL, while 22 showed marked enrichment in PTL. Among these, five characteristic pathways achieved an LDA threshold > 2.5 (Figure 4). The PTL exhibited enrichment in three pathways: Biosynthesis of terpenoids and steroids, Ribosome, and Valine, leucine and isoleucine biosynthesis. Conversely, the CJL demonstrated enrichment in Limonene and pinene degradation and Geraniol degradation. Notably, CJL showed preferential enrichment in catabolic pathways (e.g., terpenoid degradation, benzoate degradation, and fatty acid degradation) and amino acid metabolism (e.g., valine/leucine/isoleucine degradation and histidine metabolism). In contrast, PTL exhibited dominance in genetic information processing (e.g., transcription, DNA repair, and DNA replication), antibiotic biosynthesis (e.g., vancomycin resistance and streptomycin biosynthesis), and cofactor/vitamin biosynthesis (e.g., pantothenate and CoA biosynthesis).

### 3.4 Comparative analysis of rhizosphere soil metabolites in lemon trees with two types of rootstocks

**Figure 5:**
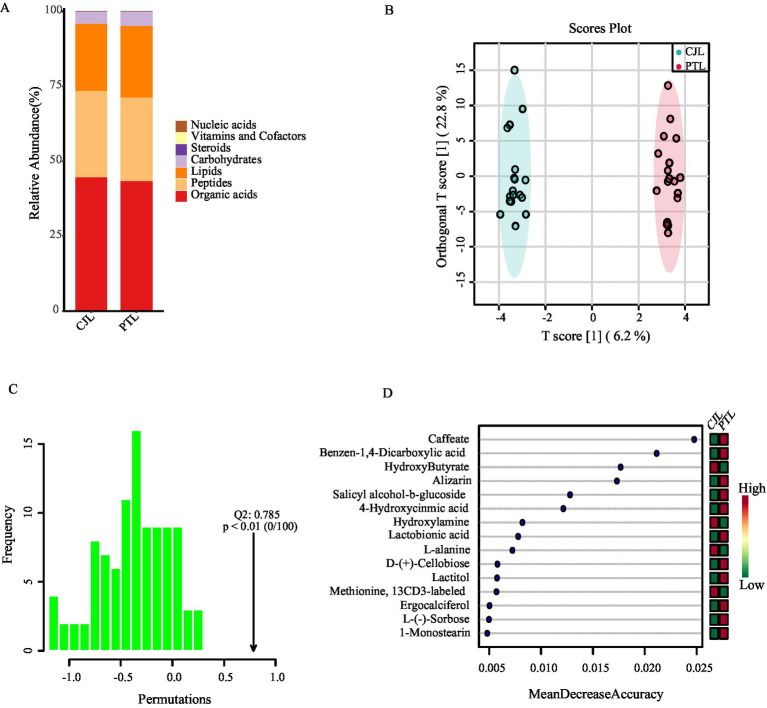
Comparative analysis of rhizosphere soil metabolites. **(A)** Relative abundance of metabolites. **(B)** OPLS-DA analysis of metabolite detection results. **(C)** Permutation test of OPLS-DA. **(D)** Random forest analysis screening for differential metabolites.

Among all rhizosphere soil samples, a total of 184 compounds were detected. By annotating all metabolites using the KEGG database (br08001), 110 biologically functional compounds were identified, primarily categorized into seven groups. Among these, organic acids exhibited the highest relative abundance, followed by peptides and lipids. The combined relative abundance of these three categories accounted for over 95% in both treatments (Figure 5A).

Orthogonal partial least squares discriminant analysis (OPLS-DA) was conducted on the rhizosphere soil metabolite detection results (Figure 5B). The results revealed that the point cloud distributions of CJL and PTL treatment samples were in distinct regions, indicating clear sample separation. Additionally, in the permutation test of OPLS-DA, the actual observed Q2 indicated by the arrow was on the right side of the random distribution (the observed value was significantly greater than the random value), with a *p*-value less than 0.01 (Figure 5C). This suggests that the discriminant effect of the OPLS-DA model was good, and there should be significantly different metabolites between the two treatments.

Random forest analysis further identified 15 discriminative metabolites (Figure 5D). Four metabolites were significantly enriched in CJL: Hydroxybutyrate, Hydroxylamine, L-Alanine, and ^13^CD_₃_-labeled Methionine. Elevations in PTL included 11 metabolites: Caffeate, Terephthalic acid (Benzen-1,4-dicarboxylic acid), Alizarin, Salicin (Salicyl alcohol-*β*-glucoside), 4-Hydroxycinnamic acid, Lactobionic acid, D-(+)-Cellobiose, Lactitol, Ergocalciferol (Vitamin D_₂_), L-Sorbose, and 1-Monostearin.

### 3.5 Integrative metagenomic and metabolomic analysis reveals rootstock-driven microbial-metabolic interactions‌

**Figure 6:**
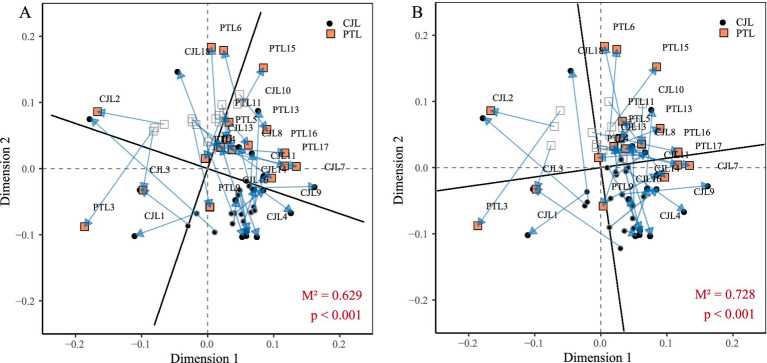
Procrustes analysis. **(A)** Species-metabolite Procrustes analysis. **(B)** Functional-metabolite Procrustes analysis.

Procrustes analysis using Bray-Curtis distances was performed to investigate relationships between rhizosphere microbial communities, functional profiles, and soil metabolites in two rootstock lemon cultivars. Overall, significant systematic concordance was observed among variations in microbial taxa composition, functional traits, and metabolite profiles (*p* < 0.001). Notably, microbial community structure exhibited stronger alignment with metabolite variations compared to functional profiles. Microbial community structure explained 37.1% of metabolite variance (M^2^ = 0.629; 1 − M^2^ = 0.371), with Dimension 1 coordinates reflecting primary associations between dominant microbial taxa and key metabolites. Both CJL and PTL samples clustered within the 0–0.2 range along this axis, indicating synchronous variation patterns in both treatments. Dimension 2 coordinates captured secondary associations involving rare microbial species and trace metabolites. CJL samples occupied the −0.1 to 0 range on this axis, suggesting antagonistic variation patterns (Figure 6A). Microbial functional profiles explained 27.2% of metabolite variance (M^2^ = 0.728; 1 − M^2^ = 0.272), with functional-metabolite covariation trends mirroring community-metabolite associations (Figure 6B).

**Figure 7:**
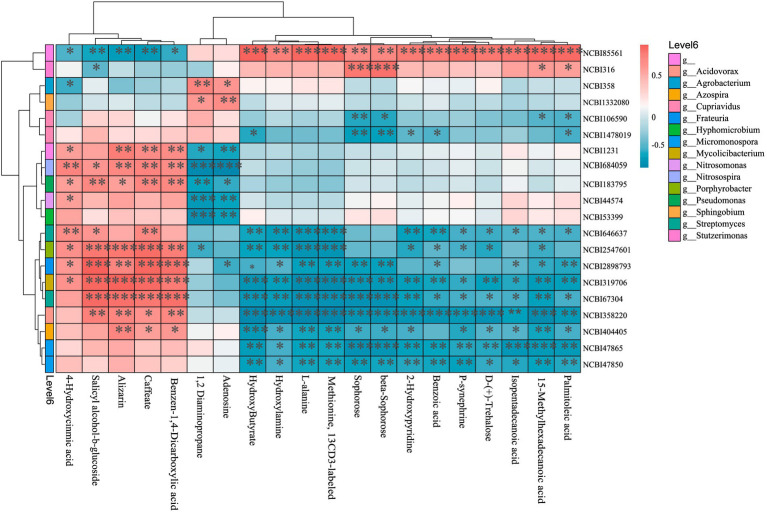
Microbe-metabolite correlation heatmap (top 20). The vertical axis represents metagenomic features (taxonomically annotated as phylum/class/genus/species where applicable). The horizontal axis denotes metabolomic features. The upper dendrogram reflects similarity clustering of metabolomic features, while the left-side dendrogram represents clustering of metagenomic features. The central heatmap illustrates pairwise correlations between the two omics datasets, with color intensity corresponding to correlation coefficients (see color scale at top right: warm colors for positive correlations, cool colors for negative correlations). NCBI85561: *EDTA-degrading bacterium* BNC1; NCBI361: *Stutzerimonas stutzeri*; NCBI358: *Agrobacterium tumefaciens*; NCBI1332080: *Sphingobium baderi*; NCBI106590: *Cupriavidus necator*; NCBI1478019: *Cupriavidus* sp. KK10; NCBI1231: *Nitrosospira multiformis*; NCBI684059: *crenarchaeotes*; NCBI183795: *Pseudomonas mediterranea*; NCBI44574: *Nitrosomonas communis*; NCBI53399: *Hyphomicrobium denitrificans*; NCBI646637: *Streptomyces* sp. M2; NCBI2547601: *Porphyrobacter* sp. YT40; NCBI2898793: *Frateuria edaphi*; NCBI319706: *Mycolicibacterium phocaicum*; NCBI67340: *Streptomyces phaeopurpureus*; NCBI358220: *Acidovorax* sp. KKS102; NCBI404405: *Azospira restricta*; NCBI47865: *Micromonospora inositola*; NCBI47850: *Micromonospora aurantiaca*.*p < 0.05; **p < 0.01; ***p < 0.001.

To further investigate microbe-metabolite interactions, a correlation heatmap was constructed using the top 20 most relevant microbes and metabolites (Figure 7). Highly correlated microbes predominantly belonged to the classes Actinomycetes (phylum Actinomycetota) and Alphaproteobacteria, Betaproteobacteria, Gammaproteobacteria (phylum Pseudomonadota), while key metabolites included amino acids, organic acids/derivatives, fatty acids, and sugars. The relative abundance of the top 15 metabolites showed positive correlations with *EDTA-degrading bacterium* BNC1 and *Stutzerimonas stutzeri*, but negative correlations with 18 soil microbes—particularly *Streptomyces* sp. M2, *Porphyrobacter* sp. YT40, *Frateuria edaphi*, *Mycolicibacterium phocaicum*, *Streptomyces phaeopurpureus*, *Acidovorax* sp. KKS102, *Azospira restricta*, *Micromonospora inositola*, and *Micromonospora aurantiaca*. Conversely, the last five metabolites exhibited a reverse correlation pattern.

**Figure 8:**
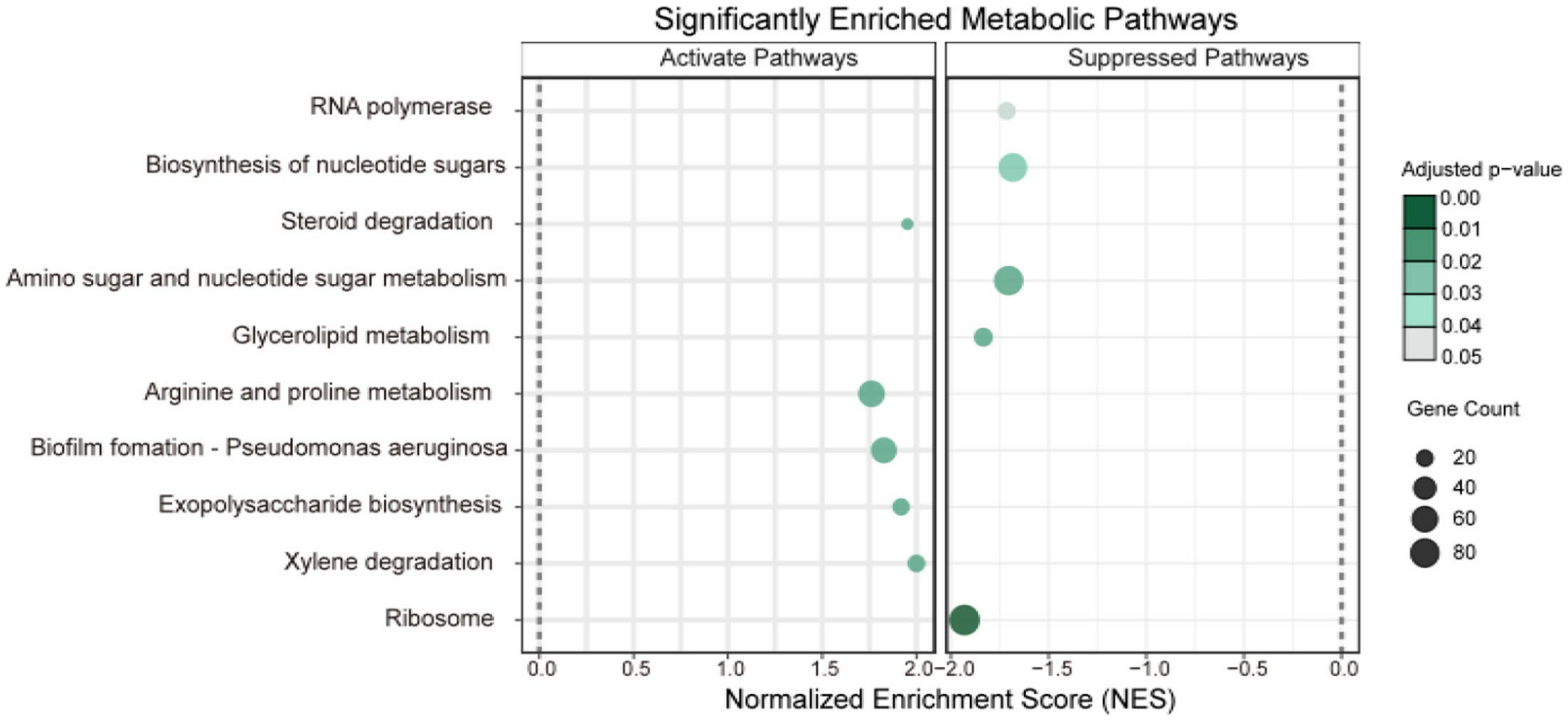
GSEA enrichment analysis bubble plot. The vertical axis represents pathways jointly enriched by the two omics datasets. The horizontal axis denotes the geneRatio (defined as the ratio of target genes enriched in a given pathway to the total number of target genes). The size of dots corresponds to the number of enriched genes, while the color intensity reflects the magnitude of the *p*-value (see color scale).

To elucidate broader biological trends and pathway alterations, Gene Set Enrichment Analysis (GSEA) was applied to all features across the two omics datasets (Figure 8). Ten significantly differentially expressed pathways (adjusted *p*-value < 0.05) were identified. Compared to PTL, the CJL treatment exhibited significant activation of: xylene degradation, exopolysaccharide biosynthesis, biofilm formation (*Pseudomonas aeruginosa*), arginine and proline metabolism, and steroid degradation. Conversely, pathways markedly suppressed in CJL included: ribosome, glycerolipid metabolism, amino sugar and nucleotide sugar metabolism, biosynthesis of nucleotide sugars, and RNA polymerase.

## 4 Discussion

### 4.1 Structural divergences in rhizosphere microbial communities across rootstock genotypes

Rhizosphere soil microorganisms constitute an indispensable factor in agricultural production, as plant growth, soil fertility, material cycling, and energy transformation are profoundly influenced by their activity (Deng et al., 2017). Crop species (Compant et al., 2019), developmental stages (Schlechter et al., 2019), root exudates (Sasse et al., 2018; Vives-Peris et al., 2020), and rhizodeposits (Tian et al., 2020) collectively shape the composition and functionality of plant-associated rhizosphere microbial communities. In this study, comparative analysis of rhizosphere microbial communities between lemon rootstocks revealed no significant difference in microbial richness, but *Poncirus trifoliate* Raf rootstock (PTL) exhibited significantly higher community evenness than *Citrus junos* ex Tanaka rootstock (CJL). This suggests that PTL-associated rhizosphere microbes are less sensitive to singular environmental stressors (e.g., pH fluctuations, nutrient variations) and possess enhanced systemic resilience (Zhang et al., 2014). Beta diversity analysis demonstrated distinct separation in microbial composition between rootstocks, indicating rootstock-specific modulation of rhizosphere microbiota, consistent with findings in barley (Bulgarelli et al., 2015), rice (Edwards et al., 2018), and common bean (Perez-Jaramillo et al., 2019). Further taxonomic profiling identified Pseudomonadota, Actinomycetota, and Thermoproteota as dominant phyla across both rootstocks. Pseudomonadota members, particularly pseudomonads, are renowned for their biocontrol potential against soil-borne pathogens (Biessy and Filion, 2021; Carrion et al., 2019). Actinomycetota contribute to plant defense by suppressing pathogens and pests, decomposing soil organic matter, mobilizing mineral nutrients, and enhancing soil enzyme activity, thereby improving rhizosphere physicochemical properties. Thermoproteota, a group of archaeal prokaryotes, serve as key contributors to carbon cycling (Xu et al., 2021; Barns et al., 1996). We identified 15 enriched microbial genera across three phyla (Actinomycetota, Pseudomonadota, and Nitrososphaerota) in the two treatments. Notably, *Pseudomonas* (class Gammaproteobacteria), *Cupriavidus* (class Betaproteobacteria), and *Burkholderia* (class Betaproteobacteria) in the CJL group, along with *Sphingobium* (class Alphaproteobacteria) in the PTL group, exhibited significant differentiation with strong effects. This divergence may reflect co-domestication processes, whereby crop cultivars selectively recruit specialized microbiomes during domestication (Escudero-Martinez and Bulgarelli, 2019). Functional annotation revealed five differentially enriched metabolic pathways. PTL rhizosphere exhibited upregulation of terpenoid and steroid biosynthesis, ribosome biogenesis, and branched-chain amino acid biosynthesis, whereas CJL microbiota specialized in limonene/pinene degradation and geraniol degradation. These microbial-derived metabolites mediate interplant communication, disease resistance, and growth promotion (Ortíz-Castro et al., 2009), with functional specificity likely determined by rootstock-dependent microbial recruitment.

### 4.2 Rootstock-specific variation in rhizosphere metabolomic profiles

Plant secondary metabolites encompass volatile and non-volatile compounds, including small molecules (phenolics, amino acids, nucleotides, sugars, terpenoids, lipids) and macromolecules (nucleic acids, polysaccharides, proteins). These metabolites regulate plant development, innate immunity (Piasecka et al., 2015), defense signaling (Isah, 2019), and environmental stress responses (Yang et al., 2018). Roots actively or passively secrete secondary metabolites into the rhizosphere, where most are rapidly metabolized by soil microbes, while residual fractions mediate interspecies interactions (Sugiyama and Yazaki, 2014). Rhizosphere metabolites originate from root exudation, microbial metabolism, and decomposition of plant/microbial biomass and soil organic matter (Cheng et al., 2018), with root exudates constituting the primary source (Song et al., 2020). Exudate composition dynamically responds to plant genotype, developmental stage, and environmental stressors (Korenblum et al., 2020). Metabolomic analysis identified 15 differentially abundant rhizosphere metabolites. CJL rhizosphere accumulated four metabolites, primarily amino acids (L-alanine, methionine) and organic acid derivatives, which enhance salt tolerance in crops such as maize (Neto et al., 2009), cucumber (Wu et al., 2012), and rice (Ghasemi et al., 2014) while modulating rhizosphere microbiota (Yuan et al., 2018; Pang et al., 2021). PTL rhizosphere showed higher abundance of 11 metabolites, predominantly sugar derivatives and organic acids. Sugar accumulation improves abiotic stress resilience (Pramanik et al., 2017; Kusale et al., 2021), while organic acids mitigate salt stress via cation chelation (Hossain et al., 2012; Adeleke et al., 2016) and rhizosphere pH modulation (Yang et al., 2010; El-Beltagi et al., 2017).

### 4.3 Functional linkages between rhizosphere microbiota and metabolites across rootstock genotypes

Elucidating how rhizosphere metabolites regulate soil-microbe-plant interactions is critical for deciphering the feedback mechanisms underlying rootstock-specific effects on plant health and crop productivity. Microbiome variations between cultivars may arise from differences in root exudates and secondary metabolites (Iannucci et al., 2017), which directly drive shifts in soil microbial composition and functionality (Pang et al., 2021). Root exudates act as primary mediators for recruiting beneficial rhizosphere microbes (Zhalnina et al., 2018), with distinct exudate profiles exerting specific impacts on rhizosphere microbiomes (Pascale et al., 2020). Studies demonstrate that roots release diverse chemicals, including sugars, amino acids, organic acids, phenolics (e.g., flavonoids), and terpenoids. These metabolites not only provide carbon substrates for microbial growth but also function as signaling molecules, attractants, or inhibitors to shape microbial communities, serving as a central hub in plant–soil-microbe interactions (Bertin et al., 2023; Baetz and Martinoia, 2014; Hu et al., 2018; De Vries et al., 2020; Zhao et al., 2020). Therefore, we investigated functional linkages between soil metabolites and microbiota across rootstock cultivars. Samples from distinct treatments exhibited highly consistent covariation patterns (*p* < 0.01) in metagenomic taxonomic composition, functional profiles, and metabolomic expression. Significant correlations between soil microbial community structure and metabolite signatures further substantiate that microbe-rhizosphere metabolite interactions constitute a key mechanism governing rhizosphere metabolic reprogramming.

This study analyzed correlated microbial taxa and metabolites, focusing on the top 20 most strongly associated pairs. Dominant microbial clades included Actinomycetes, Alphaproteobacteria, Betaproteobacteria, and Gammaproteobacteria, while primary metabolites comprised amino acids, organic acids/derivatives, fatty acids, and sugars. Correlation analyses revealed that five metabolites—salicyl alcohol-*β*-glucoside, 4-hydroxycinnamic acid, caffeate, benzene-1,4-dicarboxylic acid (a microbial degradation intermediate of anthropogenic pollutants), and alizarin (predominantly sugar/organic acid derivatives originating from roots)—exhibited significant synergism with 11 rhizobacterial species affiliated with Actinomycetota, Pseudomonadota, and Thermoproteota. This aligns with Bharti et al. (2016), demonstrating root-exuded sugars/organic acids facilitate soil microbial carbon cycling and growth promotion. Conversely, these five metabolites strongly antagonized the EDTA-degrading bacterium BNC1, a Gram-negative strain utilizing EDTA as its sole C/N source; competitive niche exclusion by sugar-stimulated actinobacterial proliferation may drive this suppression (Trivedi et al., 2020; Nortemann, 1992). In contrast, the remaining 15 metabolites—primarily microbial-derived, with minor root/exogenous contributions (e.g., methionine-13CD3, a synthetic residue)—showed inverted effects: antagonism toward the aforementioned 11 rhizobacteria, potentially mediated by bacterial interference (e.g., quorum sensing quenching; Trivedi et al., 2020) or dual-regulatory compounds like benzoate derivatives (Meng et al., 2023), while synergistically promoting BNC1. This BNC1-specific enhancement may arise from its selective substrate utilization (e.g., vitamin stimulation by biotin/folate; Nortemann, 1992), though its soil metabolic traits remain poorly characterized.

Subsequent integration of the top 15 metabolite contributors identified by Random Forest analysis with differentially abundant microbial taxa from LEfSe (LDA > 3.5), followed by exclusion of exogenous compounds and non-significant correlations (*p* > 0.05), revealed distinct biological associations: PTL rootstocks exhibited positive correlations between eight metabolites (predominantly lipids, carbohydrates, and organic acids) and seven rhizosphere microbial taxa (Table 1), while CJL rootstocks demonstrated synergistic relationships involving three metabolites (hydroxybutyrate, L-alanine, and hydroxylamine) and seven rhizospheric microbes (Table 2). Elevated root secretion of amino acids, nucleotides, and long-chain organic acids (LCOAs) has been shown to recruit beneficial Proteobacteria, Streptomyces, and Firmicutes, reshaping rhizosphere microbiomes into a “defense biome” that enhances host stress resilience (Yuan et al., 2018; Bakker et al., 2018; Liu and Brettell, 2019; Liu et al., 2020; Williams and de Vries, 2020). Similar mechanisms are documented in *Zea mays* (Ahmad et al., 2011; Cotton et al., 2019), *Cirsium* (Verbeek and Kotanen, 2019), and *Spartina alterniflora* (Yang et al., 2019). Conversely, root-derived secondary metabolites can selectively promote or inhibit specific microbial taxa (Holmer et al., 2017; Hu et al., 2018; Voges et al., 2019).

**Table 1:** Correlations between relative abundance of root-enriched specific microorganisms and rhizosphere metabolites under PTL treatment.

Microorganism names	Ergocalciferol	Lactitol	Lactobionic acid	4-Hydroxycinmic acid	1-Monostearin	Caffeate	D-(+)-Cellobiose	Salicyl alcohol-b-glucoside
*Aminobacter* sp. MDW-2	0.60^**^	0.20	0.25	0.50^*^	0.23	0.32	0.01	0.32
*Ensifer adhaerens*	0.45	0.53^*^	0.66^**^	0.51^*^	0.40	0.69^***^	0.46	0.66^**^
*Mycolicibacterium phocaicum*	0.37	0.41	0.58^*^	0.56^*^	0.32	0.77^***^	0.40	0.79^***^
*Nitrosospira multiformis*	0.65^**^	0.56^*^	0.52^*^	0.65^**^	0.42	0.64^**^	0.27	0.55^*^
*Pseudomonas mediterranea*	0.66^**^	0.56^*^	0.52^*^	0.50^*^	0.57^*^	0.62^**^	0.52^*^	0.62^**^
*Pseudonocardia dioxanivorans*	0.46	0.51^*^	0.53^*^	0.67^**^	0.34	0.70^***^	0.41	0.67^**^
*Sphingobium* sp. TKS	0.45	0.46	0.59^**^	0.40	0.47	0.66^**^	0.53^*^	0.65^**^

Asterisks denote significant differences (^*^p < 0.05, ^**^p < 0.01, ^***^p < 0.001).

**Table 2:** Correlations between relative abundance of root-enriched specific microorganisms and rhizosphere metabolites under CJL treatment.

Microorganism names	HydroxyButyrate	L-alanine	Hydroxylamine
*Azoarcus* sp. DD4	0.46	0.52^*^	0.43
*Chelativorans oligotrophicus*	0.50^*^	0.50^*^	0.45
*Cupriavidus oxalaticus*	0.58^*^	0.59^**^	0.55^*^
*Metapseudomonas furukawai*i	0.63^**^	0.70^***^	0.63^**^
*Metapseudomonas lalkuanensis*	0.33	0.46	0.39
*Pseudomonas putida*	0.64^**^	0.68^***^	0.63^**^

*p < 0.05; **p < 0.01; ***p < 0.001.

### 4.4 Interaction patterns among root systems, metabolites, and microbiota across rootstock genotypes

**Figure 9:**
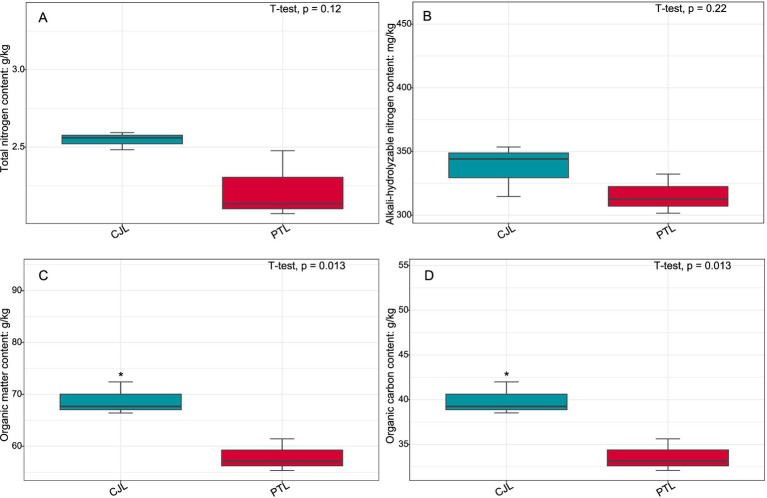
Contents of total nitrogen, alkali-hydrolyzable nitrogen, organic matter, and organic carbon in rhizosphere soils of lemon under two rootstock genotypes. **(A)** Total nitrogen content; **(B)** Alkali-hydrolyzable nitrogen content; **(C)** Organic matter content; **(D)** Organic carbon content. Asterisks denote significant differences (^*^p < 0.05; ^**^p < 0.01).

**Figure 10:**
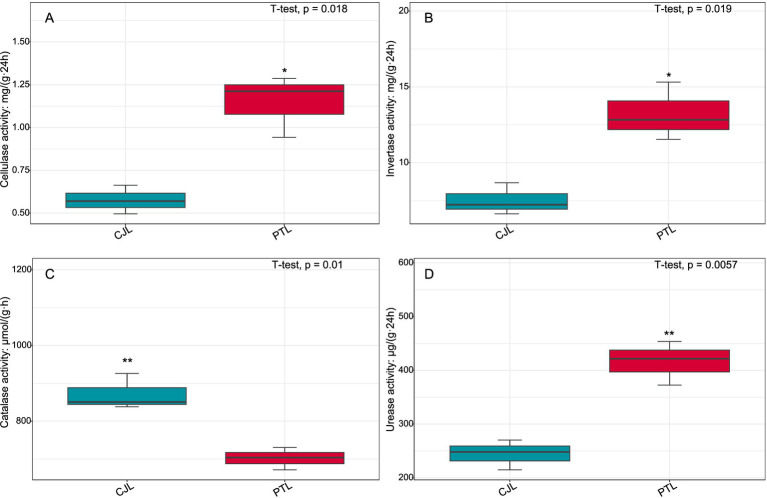
Activities of cellulase, sucrase, catalase, and urease in rhizosphere soils of lemon grafted onto two rootstock genotypes. **(A)** Cellulase activity; **(B)** Sucrase activity; **(C)** Catalase activity; **(D)** Urease activity. *p < 0.05; **p < 0.01.

**Figure 11:**
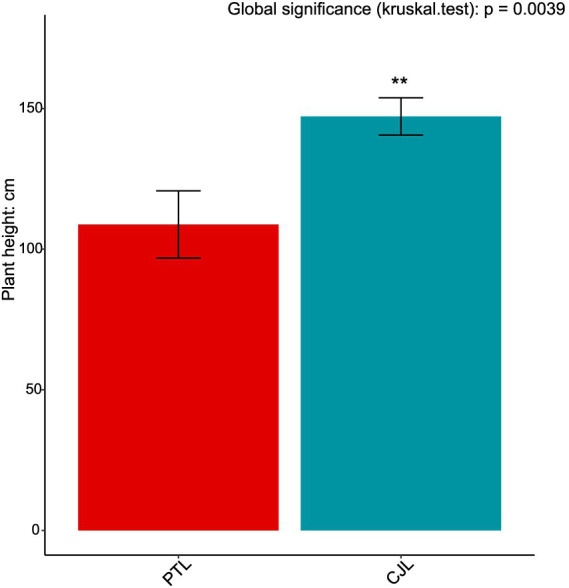
Plant height of lemon grafted onto two rootstock genotypes. **p < 0.01.

To further investigate biological trends in the soil microbe-root exudate system, enrichment analysis was performed on all features from both omics datasets, identifying 10 significantly differentially expressed pathways (*p* < 0.05). Concurrently, key rhizosphere soil physicochemical properties were quantified: total nitrogen, alkali-hydrolyzable nitrogen, organic matter, and organic carbon content (Figure 9); critical enzyme activities including cellulase, sucrase, catalase, and urease (Figure 10); and experimental tree height (Figure 11).

Three pathways related to soil microorganism-root exudate interactions were enriched in PTL: Glycerolipid metabolism, Amino sugar and nucleotide sugar metabolism, and Biosynthesis of nucleotide sugars, primarily associated with energy metabolism, carbohydrate metabolism, and secretion regulation. Glycerolipid metabolism serves as a core pathway in lipid metabolism, involving triglyceride (TG) breakdown and synthesis to provide energy reserves and biomembrane construction. *Pseudomonas mediterranea*, significantly enriched in this study, is a keystone taxon in glycerolipid metabolism (Solaiman et al., 2005). Studies indicate *Pseudomonas* promotes rhizosheath formation under drought stress while its metabolites (e.g., IAA) directly stimulate lateral root meristem proliferation, increasing root surface area and enhancing water/nutrient uptake efficiency (Xu et al., 2025). The metabolite 1-Monostearin, an intermediate in glycerolipid metabolism, is primarily synthesized and secreted by plant roots as a precursor for triacylglycerol (TAG) synthesis (Liu et al., 2007). Here, 1-Monostearin showed significant positive correlation with *P. mediterranea* abundance, suggesting PTL may recruit *P. mediterranea* by secreting 1-Monostearin to activate glycerolipid metabolism, thereby enhancing root functionality and adaptation to arid/oligotrophic environments. Amino sugar and nucleotide sugar metabolism regulates microbial necromass formation and soil organic carbon sequestration. Associated metabolites included Lactitol, Lactobionic acid, and D-(+)-Cellobiose (microbially derived), with key microbes *Ensifer adhaerens* and *Sphingobium* sp. TKS. *E. adhaerens* utilizes rhizospheric cellobiose (D-(+)-Cellobiose) as a carbon source to activate nucleotide sugar metabolism (e.g., UDP-glucose synthesis), secreting exopolysaccharides (EPS), siderophores, salicylic acid (SA), and IAA to enhance root defense and stimulate lateral root proliferation (Zheng T. et al., 2021; Hu et al., 2024). *Sphingobium* sp. TKS degrades *γ*-HCH as its sole carbon source for organic pollutant decomposition (Tabata et al., 2016). It catabolizes lactobionic acid to mannose-6-phosphate, entering nucleotide sugar metabolism (GDP-mannose pathway). Resultant EPS enhances soil aggregation, improving root oxygenation, while mannosylated signaling molecules induce plant disease resistance genes (Zheng T. et al., 2021). Notably, root-secreted caffeate and salicyl alcohol-*β*-glucoside showed extremely significant positive correlations with both *E. adhaerens* and *Sphingobium* sp. TKS (Table 1), indicating PTL recruits these taxa via exudation to activate amino/nucleotide sugar metabolism, thereby modulating SOC sequestration, root defense, and lateral root development. Biosynthesis of nucleotide sugars operates inversely to nucleotide sugar metabolism. This pathway was present in *E. adhaerens*, *P. mediterranea*, *Mycolicibacterium phocaicum*, *Pseudonocardia dioxanivorans*, and *Sphingobium* sp. TKS, all exhibiting extremely significant correlations with caffeate and salicyl alcohol-β-glucoside (Table 1). This implies PTL recruits these microbes to activate nucleotide sugar biosynthesis, providing biosynthetic precursors for beneficial microbiota proliferation while balancing metabolic flux. Soil enzyme assays (Figure 10) revealed significantly upregulated cellulase, sucrase, and urease activities in PTL, corresponding to the enrichment of glycerolipid metabolism, amino/nucleotide sugar metabolism, and nucleotide sugar biosynthesis pathways.

Three pathways related to soil microorganism-root exudate interactions were enriched in CJL: Exopolysaccharide biosynthesis, Arginine and proline metabolism, and Biofilm formation—*Pseudomonas aeruginosa*, primarily associated with stress resistance, cellular membrane homeostasis, and signal transduction. Exopolysaccharide biosynthesis enhances stress tolerance through protective biofilm formation via extracellular polymeric substance (EPS) secretion, which isolates roots from external stresses while improving water retention and soil structural stability to sustain plant growth (Nadeem et al., 2021). *Pseudomonas* strains synthesize EPS (e.g., alginate) (Zhao et al., 2017); notably, *Pseudomonas putida* identified in this study exhibited extremely significant positive correlations with key metabolites L-alanine, 3-Hydroxybutyrate, and Hydroxylamine (Table 2). While 3-Hydroxybutyrate (a critical energy/carbon source) and Hydroxylamine (regulating nitrogen metabolism) are primarily rhizosphere microbial products, L-alanine originates from roots and provides nitrogen/carbon skeletons for microbial metabolism. This suggests CJL may recruit *P. putida* via L-alanine exudation to activate exopolysaccharide biosynthesis, thereby enhancing root stress tolerance—a potential mechanism underlying the strong alkali resistance of *Citrus junos* (CJ). EPS also mitigates soil acidification while promoting organic matter decomposition and nutrient availability (Kumar et al., 2021; Chen et al., 2023), consistent with CJL’s significantly higher organic matter and organic carbon content (Figures 9C,D). Amino acids serve as essential nitrogen sources and signaling factors. Proline (Pro) regulates stress metabolism by maintaining cytosolic homeostasis and reactive oxygen species scavenging (Singh et al., 2016), whereas L-arginine participates in cell division, DNA condensation, membrane stabilization, hormone signaling, and stress responses (Agudelo-Romero et al., 2013). The significant activation of arginine and proline metabolism in CJL aligns with studies demonstrating that plant growth-promoting rhizobacteria elevate osmoprotectants including proline, soluble sugars, and free amino acids (Ilyas et al., 2020; Khan and Singh, 2021). *P. putida* enriched in CJL participates in amino acid metabolism in *Zea mays* (Sandhya et al., 2010) and *Triticum aestivum* (Khan and Singh, 2021) rhizospheres, potentially functioning via: (a) direct engagement in exopolysaccharide biosynthesis through arginine/proline metabolism to enhance root stress tolerance, and (b) modulation of other microbiota growth via secretion systems. CJL’s elevated catalase activity (Figure 10C) further corresponds with upregulated arginine/proline metabolism. Biofilm formation enables rhizospheric microbial colonization and community assembly on root surfaces (Haggag and Timmusk, 2010), with continuous biofilm cycling conferring environmental adaptability (Koerdt et al., 2010). Although the Biofilm formation—*P. aeruginosa* pathway was activated in CJL, *P. aeruginosa* relative abundance remained unchanged, likely due to its c-di-GMP-mediated enhancement of existing biofilm formation through alginate and extracellular DNA secretion without triggering bacterial proliferation (Tsiry et al., 2015)—consistent with upregulated exopolysaccharide biosynthesis herein. Plant height analysis (Figure 11) revealed CJL significantly surpassed PTL, a difference primarily attributed to rootstock characteristics (Li et al., 2019) rather than rhizobacterial influences, for which effects on plant height remain poorly documented.

Integrated analysis revealed that CJL treatment exhibited significantly higher soil organic matter, organic carbon content, catalase activity, and plant height compared to PTL, while PTL demonstrated significantly elevated cellulase, sucrase, and urease activities. No significant differences were observed in total nitrogen or alkali-hydrolyzable nitrogen between treatments. The root-metabolite-microbe interaction patterns in PTL emerged as follows: (1) Root-secreted 1-Monostearin potentially activates glycerolipid metabolism in *Pseudomonas mediterranea*, recruiting this bacterium to enhance root functionality and adaptation to arid/oligotrophic environments; (2) Root-exuded caffeate and salicyl alcohol-*β*-glucoside may recruit *Sphingobium* sp. TKS and *Ensifer adhaerens* to stimulate amino sugar and nucleotide sugar metabolism, thereby regulating rhizosphere soil organic carbon sequestration, root defense, and lateral root proliferation; (3) These same exudates recruit *Ensifer adhaerens*, *Pseudomonas mediterranea*, *Mycolicibacterium phocaicum*, *Pseudonocardia dioxanivorans*, and *Sphingobium* sp. TKS to activate nucleotide sugar biosynthesis, providing biosynthetic precursors for beneficial microbiota while balancing metabolic flux. In CJL, the predominant pattern involves: (1) Root-secreted L-alanine recruits *Pseudomonas putida* to activate exopolysaccharide biosynthesis, enhancing root stress tolerance—a potential mechanism underlying *Citrus junos*’ alkali resistance; (2) L-alanine-recruited *P. putida* engages arginine and proline metabolism to participate directly in exopolysaccharide biosynthesis, further promoting root stress resilience. Although plant microbiome structures and dynamics are well-documented (Pang et al., 2021), microbial contributions to host rhizosphere metabolomes remain poorly understood. Rhizosphere microbiomes critically regulate plant growth and health (Berendsen et al., 2012), with specific bacteria intimately interacting with roots to induce functional modifications in root exudate composition (Rolfe et al., 2019; Baetz and Martinoia, 2014; Huang et al., 2018). Plants may also perceive microbiome-derived molecules through chemical recognition systems, triggering signal transduction networks that alter gene activity and metabolite accumulation (Tidke et al., 2018). While these interaction patterns provide crucial insights for agricultural productivity enhancement, the extraordinary complexity of microbial diversity and rhizosphere metabolome composition necessitates further validation and mechanistic exploration of the proposed models.

## 5 Conclusion

The rhizosphere microbial community structures of lemon trees grafted onto two distinct rootstocks exhibited significant divergence, with three phyla (Actinomycetota, Pseudomonadota, and Nitrososphaerota) comprising 15 differentially enriched genera showing marked effects—particularly *Pseudomonas*, *Cupriavidus*, and *Burkholderia* in CJL, and *Sphingobium* in PTL. Correspondingly, rhizosphere metabolite profiles differed substantially between rootstocks: among 15 key differential metabolites identified, 4 were significantly more abundant in CJL, while the remaining 11 dominated in PTL. Strong correlations emerged between differential microbial abundance and metabolite levels, with 10 significantly altered pathways implicated in energy homeostasis and environmental adaptation. CJL soils demonstrated significantly higher organic matter/organic carbon content, catalase activity, and plant height, whereas PTL exhibited elevated cellulase, sucrase, and urease activities—though total and alkali-hydrolyzable nitrogen showed no inter-treatment differences. The root-metabolite-microbe interaction paradigm in PTL involved: (1) root-secreted 1-Monostearin activating *Pseudomonas mediterranea*’s glycerolipid metabolism to enhance drought/oligotrophic adaptation; (2) caffeate and salicyl alcohol-β-glucoside recruiting *Sphingobium* sp. TKS and *Ensifer adhaerens* to stimulate amino/nucleotide sugar metabolism, thereby modulating carbon sequestration, root defense, and lateral root proliferation; (3) these exudates further assembling *Ensifer adhaerens*, *Pseudomonas mediterranea*, *Mycolicibacterium phocaicum*, *Pseudonocardia dioxanivorans*, and *Sphingobium* sp. TKS to orchestrate nucleotide sugar biosynthesis, supporting microbiota proliferation while balancing metabolic flux. In CJL, L-alanine secretion recruited *Pseudomonas putida* to activate exopolysaccharide biosynthesis—a plausible mechanism underlying *Citrus junos*’ alkali tolerance—with *P. putida* concurrently engaging arginine/proline metabolism to reinforce this stress-resistance pathway. This study elucidates rootstock-driven modulation of lemon tree rhizosphere microbiomes and metabolomes, uncovering their interaction networks and affirming rootstocks’ pivotal role in shaping rhizosphere microecosystems. These findings provide actionable insights for rootstock selection and beneficial microbiota screening to enhance agricultural productivity.

## Data Availability

The data presented in the study are deposited in the China National Center for Bioinformation (https://www.cncb.ac.cn/) repository, accession number CRA029952.

## Supplementary material

SUPPLEMENTARY Table 1 Dataset for quality assessment results of metagenomic sequencing data.

SUPPLEMENTARY Table 2 Dataset for soil microbial alpha diversity analysis results.

SUPPLEMENTARY Table 3 Dataset for beta diversity analysis (Bray-Curtisdistancematrix).

SUPPLEMENTARY Table 4 Dataset for LEfSe analysis results of rhizosphere microbial differentials.

SUPPLEMENTARY Table 5 Dataset for microbial functional traits LEfSe analysis results.

SUPPLEMENTARY Table 6 Dataset characterizing discriminatory biomarkers from OPLS-DA analysis of untargeted metabolomic profiling.

SUPPLEMENTARY Table 7 Dataset of microbe-metabolite correlations based on Spearman’s rank analysis.

SUPPLEMENTARY Table 8 Dataset of microbe-metabolite correlations with significance validation (p-value).

SUPPLEMENTARY Table 9 Dataset for GSEA-based KEGG pathway enrichment analysis.

SUPPLEMENTARY FIGURE 1 Root system scanning images of two rootstock types.

SUPPLEMENTARY FIGURE 2 Root transverse sections of two rootstock types (paraffin sections).
