# Supplementary figures and images for: Poncirus trifoliata vs. Citrus junos rootstocks: reshaping lemon rhizosphere microecology through microbial and metabolic reprogramming
Source: Front Microbiol. 2025 Sep 24;16:1650631. doi: 10.3389/fmicb.2025.1650631 (PMC12505669; doi:10.3389/fmicb.2025.1650631)

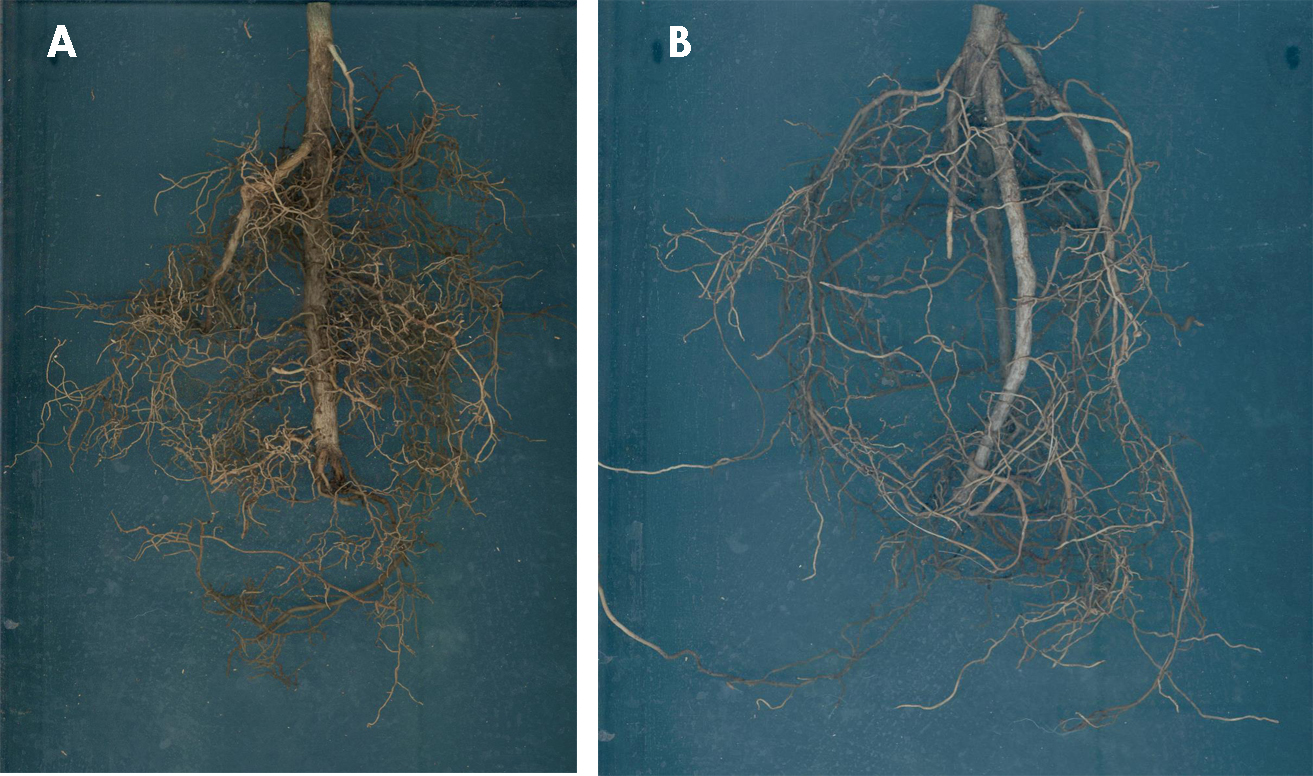

Supplement: SUPPLEMENTARY FIGURE 1 — Root system scanning images of two rootstock types. [file Image_1.jpeg]

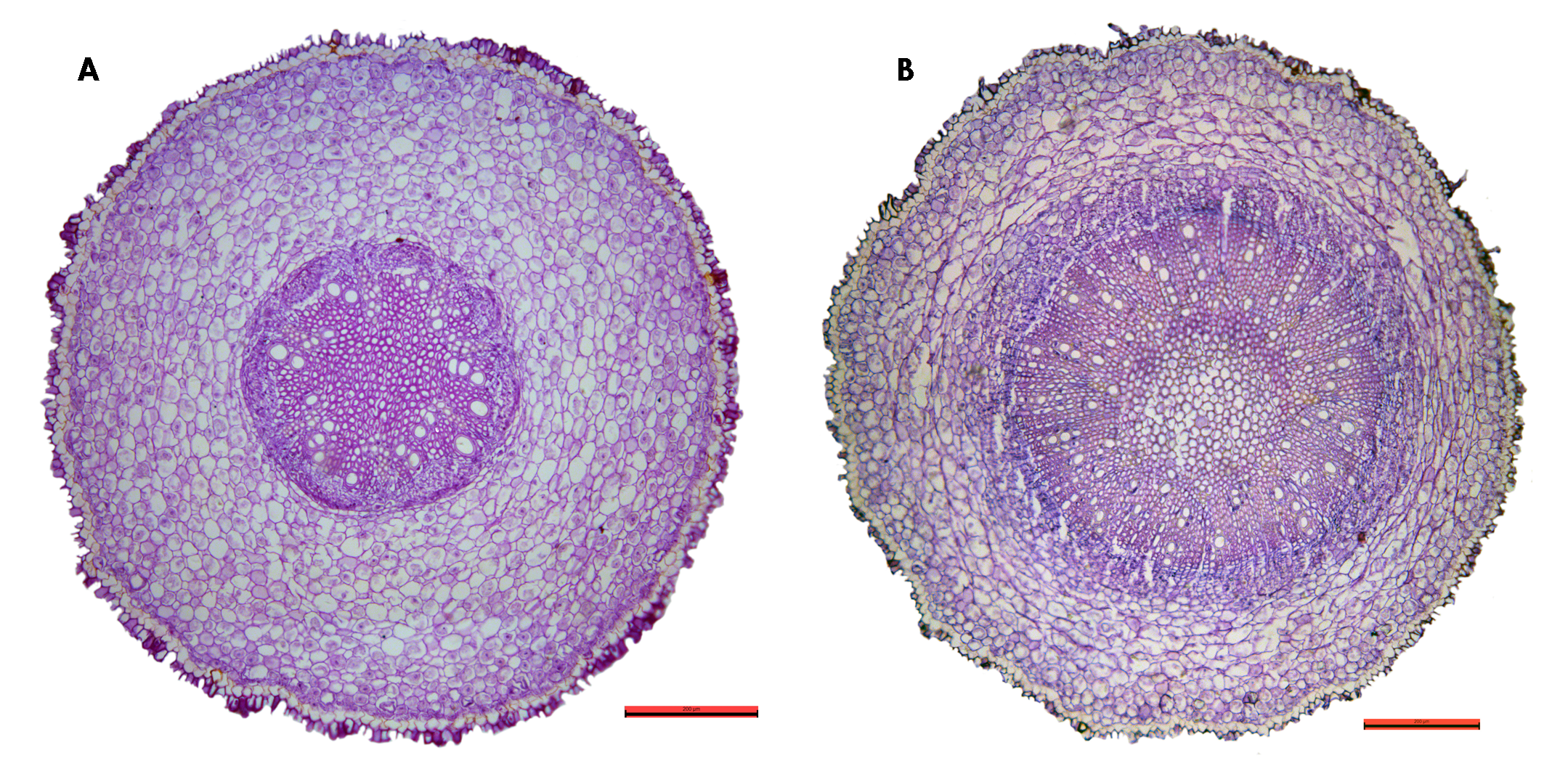

Supplement: SUPPLEMENTARY FIGURE 2 — Root transverse sections of two rootstock types (paraffin sections). [file Image_2.tif]
